# Tumor protein P63 Regulated 1 contributes to inflammation and cell proliferation of cystitis glandularis through regulating the NF-кB/cyclooxygenase-2/prostaglandin E2 axis

**DOI:** 10.17305/bjbms.2021.6763

**Published:** 2022-01-07

**Authors:** Tao Hong, Songzhe Piao, Liangxue Sun, Yiran Tao, Mang Ke

**Affiliations:** 1 Department of Urology, Taizhou Hospital of Zhejiang Province, Shaoxing University, Linhai, Zhejiang, China; 2 Department of Urology, Taizhou Hospital of Zhejiang Province affiliated to Wenzhou Medical University, Linhai, Zhejiang, China

**Keywords:** Tumor protein P63 regulated 1, inflammation, proliferation, cystitis glandularis, NF-КB/cyclooxygenase 2/prostaglandin E2

## Abstract

Cystitis glandularis is characterized by chronic inflammation and hyperproliferation of the bladder mucosa, and it contributes to the progression of bladder adenocarcinoma. Tumor Protein P63 Regulated 1 (TPRG1) is related to cellular inflammatory response, and dysregulation of TPRG1 in tumor tissues is associated with tumor recurrence. The effect of TPRG1 on cystitis glandularis was investigated in this study. Firstly, bladder specimens were isolated from patients with cystitis glandularis and *Escherichia coli-*induced cystitis rat. Expression of TPRG1 was found to be up-regulated in the bladder specimen. Moreover, adeno-associated virus (AAV)-mediated silencing of TPRG1 was injected into rat, and data from hematoxylin and eosin (H & E) staining showed that injection with AAV-shTPRG1 ameliorated *E. coli-*induced histological changes in bladder tissues of rats and suppressed the inflammatory response. Secondly, TPRG1 was also increased in primary cystitis glandularis cells. Knockdown of TPRG1 decreased cell proliferation and suppressed the migration of primary cystitis glandularis cells. Thirdly, cyclooxygenase-2 (COX-2) was up-regulated in the bladder specimens isolated from patients with cystitis glandularis and *E. coli-*induced cystitis rat. Injection with AAV-shTPRG1 reduced protein expression of COX-2, p65 and prostaglandin E2 (PGE2) in the bladder specimen. Finally, interference of COX-2 attenuated TPRG1 over-expression-induced increase in cell proliferation and migration in the primary cystitis glandularis cells. In conclusion, TPRG1 promoted inflammation and cell proliferation of cystitis glandularis through activation of NF-КB/COX2/PGE2 axis.

## INTRODUCTION

Cystitis glandularis, a metaplastic mass of bladder mucosa, shows histopathologic appearance with submucosal urothelium and glandular metaplasia of the bladder mucosa [1]. Cystitis glandularis, a chronic inflammatory disorder, is characterized by hyperproliferation in the bladder mucosa [1], and commonly identified in urothelial diseases and adenocarcinoma [2]. Cystitis glandularis was found to be associated with development and progression of bladder carcinoma [3], and the incidence is increasing recently [4]. Therefore, elucidation of pathogenesis is essential for the effective intervention.

Tumor Protein P63 Regulated 1 (TPRG1) was found to be differentially expressed in tumor tissues including breast cancer [5] and HPV-associated oropharyngeal squamous cell carcinoma [6]. Chromosomal rearrangement of TPRG1 in ordinary lipoma tissues might be associated with the tumorigenesis [7]. TPRG1 was associated with inflammatory pathways and stimulates inflammatory responses [8]. TPRG1, as an immune-related gene, was correlated with tumor recurrence of stage Ia-b lung cancer [9]. Moreover, microarray analysis showed that TPRG1 was up-regulated in bladder mucosa isolated from cystitis glandularis patients [10]. However, the effects of TPRG1 on cell proliferation and inflammation in cystitis glandularis have not been reported yet.

Chronic inflammation is a major risk factor for cystitis glandularis [11] and NF-КB signaling. This is important for secretion of proinflammatory cytokines, was activated in *Escherichia coli -*induced rat model with cystitis glandularis [12]. In addition, TPRG1 could promote the activation of NF-КB signaling [10]. Therefore, TPRG1 might regulate cystitis glandularis through modulation of NF-КB signaling. In this study, expression of TPRG1 in bladder specimens isolated from patients with cystitis glandularis was determined first. *E. coli*-induced rat model with cystitis glandularis was then established and the primary cystitis glandularis cells were isolated. The effects of TPRG1 on cell proliferation and inflammation were investigated. The present study might provide a novel therapeutic target for cystitis glandularis.

## MATERIALS AND METHODS

### Human bladder specimen

A total of 18 patients with cystitis glandularis were recruited at Taizhou Hospital of Zhejiang Province affiliated to Wenzhou Medical University from 2015 to 2018. Patients with bladder carcinoma, tuberculosis, and vesical calculi were excluded. Biopsy of bladder tissues was used to diagnose the patients. The bladder specimens were obtained from the 18 patients and served as the cystitis glandularis (CG) group. Normal bladder specimens were obtained from 8 patients undergoing transurethral bladder tumor resection, and then served as normal control. The normal bladder specimens were obtained approximately 10 cm from the patient’s tumor lesion and observed by 2 pathologists without metastasis. All the patients provided written informed consents, and this study was approved by Taizhou Hospital of Zhejiang Province affiliated to Wenzhou Medical University. The clinical information of the patients was shown in Table 1. Written informed consent was obtained from a legally authorized representative(s) for anonymized patient information to be published in this article.

**TABLE 1:**
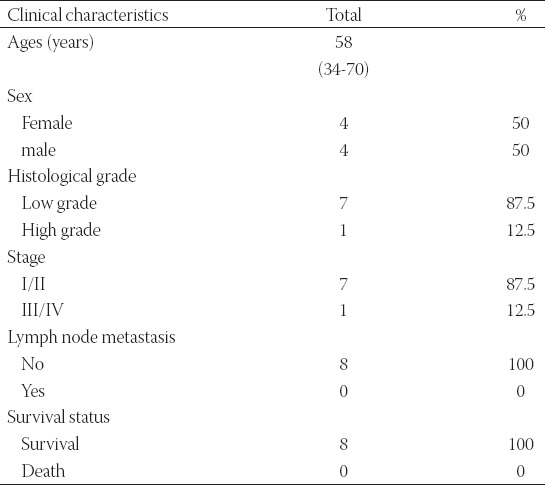
Clinical characteristics of the patients

### Animal model

The experiments were approved by Taizhou Hospital of Zhejiang Province and in accordance with those of the 1964 Helsinki Declaration and its later amendments for ethical research involving human subjects. A total of 40 female Sprague–Dawley rats (200-250 g weight and 6-8 weeks old) were purchased from the Slack Experimental Animal Company (Shanghai, China), and randomized into four groups: sham (n = 10), CG (n = 10), CG with AAV-shTPRG1 (n = 10) and CG with AAV-shNC (n = 10). Rats in the CG group were received 0.5 mL of *E. coli* through intravehicular infusion 3 times a week for 2 months. Rats in the sham group were raised under standard conditions for 2 months. Rats in the CG with AAV-shTPRG1 (n = 10) and CG with AAV-shNC (n = 10) groups were received AAV-shTPRG1 and AAV-shNC viral solution (GeneChem Technology Company, Shanghai, China) for successive 7 days. One day after the last treatment, all the rats were anesthetized, and the bladder tissue specimens were harvested.

### Immunohistochemical and histopathological analysis

Human bladder specimens were immersed in 4% paraformaldehyde, embedded in paraffin, and sliced into 4 mm sections. The sections were then dewaxed and hydrated and incubated in 3% H_2_O_2_. Following incubation in Tris-EDTA buffer containing 0.05% Tween 20 and block in 4% dry milk, the tissues were incubated with specific antibody against TPRG1 (1:80; Abcam, Cambridge, MA, USA) and COX-2 (1:50; Abcam). The sections were then treated with peroxidase-labeled goat anti-rabbit IgG secondary antibody (Abcam), and observed under microscope (Olympus, Tokyo, Japan). Bladder specimens were isolated from the rats, and also sliced into 4 mm sections. The sections were stained with hematoxylin and eosin (Sigma-Aldrich, San Francisco, CA, USA) and observed under the microscope.

### Enzyme linked immunosorbent assay (ELISA)

Bladder specimens were isolated from the rats and then lysed in RIPA lysis buffer (Beyotime, Shanghai, China). Supernatants were then harvested followed by centrifugation at 12,000 g. Protein concentration of supernatants was measured by BCA kit (Applygen, Beijing, China). Levels of Tumor Necrosis Factor Alpha (TNF-α), interleukin (IL)-6, and IL-1β were determined using ELISA kits (ExCell Biology, Inc., Shanghai, China). Serum level of Prostaglandin E2 (PGE2) was also determined using ELISA kit (ExCell Biology, Inc.).

### Immunofluorescence

The bladder sections were firstly incubated with Proteinase K and 0.3% H_2_O_2_, and then incubated with Tris-EDTA buffer (pH 9.0). Sections were blocked in 4% dry milk, and then double-stained with specific antibodies against NF-κB p65 (1:80; Abcam, Cambridge, UK) and COX-2 (1:50; Abcam). The sections were then incubated with fluorescein isothiocyanate-conjugated goat anti-rabbit IgG Alexa Fluor 488 ­secondary antibody (1:100; Abcam) or fluorescein isothiocyanate-conjugated goat anti-mouse IgG Alexa Fluor 568 (1:100; Abcam) and counterstained with 4’,6-diamidino-2-phenylindole (DAPI) before measurement under LMS880 laser scanning microscope (Zeiss, Oberkochen, Germany). The fluorescence intensity was analyzed by Image J software.

### Cell culture and transfection

Primary cystitis glandularis cells were isolated from the fresh bladder tissues isolated from the patients by adhering to the glandular cystitis tissue block. Briefly, the fresh bladder tissues were sliced first into 10-15 pieces with scissors. The pieces (1 piece per well) were then distributed into 6-well plates, and cultured in Dulbecco’s modified Eagle’s medium (Invitrogen, Carlsbad, CA, USA) supplemented with 10% fetal bovine serum (Invitrogen) and penicillin-streptomycin (Invitrogen) at 37′. The medium was changed 3 times weekly, and the cells were passaged using TrypLE™ Express (Thermo Fisher Scientific, Waltham, MA, USA) when the epithelial cells grown from the primary explants. Cells isolated from the normal control patients were served as pNCs, and cells isolated from the CD patients were served as pCGs. Cells were then used for the functional assays. The pCGs were transfected with pcDNA-TPRG1, shTPRG1 or the negative controls (NC and shNC) (GeneChem Technology Company) through Lipofectamine 2000 (Invitrogen). The pCGs were also cotransfected with pcDNA-TPRG1 and siCOX2 through Lipofectamine 2000 (Invitrogen).

### Quantitative reverse transcription polymerase chain reaction (qRT-PCR)

Bladder tissues or primary cystitis glandularis cells were lysed in TRIzol kit (Invitrogen) to isolate RNAs, and the RNAs were then synthesized into cDNAs. PreTaq II kit (Takara, Dalian, Liaoning, China) was used for the qRT-PCR analysis with following primers in Table 2. The mRNA expression was normalized to GAPDH.

**TABLE 2:**
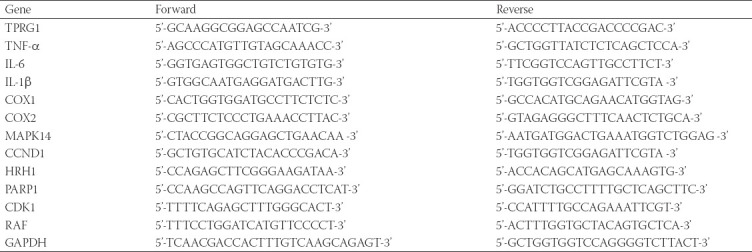
Primer sequences

### Cell proliferation assays

The pCGs were seeded in a 96-wells plate for 24, 48 or 72 hours, and then incubated with 10 μL CCK8 reagent (Beyotime) for 2 hours. Absorbance at 450 nm was detected by microplate reader (Thermo Fisher Scientific). For EdU staining, cells were seeded in a 48-wells plate, and then incubated with 200 μL EdU (Beyotime) for 2 hours. The cells were fixed with 4% paraformaldehyde and permeabilized with 0.5% Triton X-100. Following incubation with Apollo Staining reaction liquid, cells were counterstained with DAPI before measurement under Olympus ZKX53 microscope (Olympus, Tokyo, Japan).

### Cell migration assay

Cells in serum-free medium were planted into the upper champers of Transwell chambers (Corning Incorporated, Corning, NY, USA), and medium with 10% fetal bovine serum were planted into the lower champers. Twenty-four hours later, cells in the upper chamber were removed, and the cells in the lower chamber were stained with crystal violet. Cells were observed under the microscope (Olympus).

### Western blot

Protein samples isolated from bladder tissues and cells were separated by 10% SDS-PAGE and transferred onto nitrocellulose membrane. The membranes were blocked in 5% bovine serum albumin and probed with specific antibodies: anti-TPRG1 and anti-COX-2 (1:1500, Abcam), anti-TNF-α and anti-IL-6 (1:2000, Abcam), anti-IL-1β and anti-β-actin (1:2500, Abcam), anti-p65 and anti-PGE2 (1:3000, Abcam). A Cell Nuclear and Cytoplasmic Protein Extraction Kit (Beyotime) was used to isolate nuclear and cytoplasmic proteins from the pCGs, and the proteins were also separated and transferred. The membranes were also probed with specific antibodies: anti-p65 and anti-β-tubulin (1:3500, Abcam), anti-Histone H3 (1:4000, Abcam). Following incubation with horseradish peroxidase-conjugated secondary antibody (1:4500, Abcam) and tetramethylbenzidine, the immunoreactivities were visualized using enhanced chemiluminescence (Sigma-Aldrich).

### Statistical analysis

All the data with at least triple replicates were expressed as mean ± SEM, and analyzed by student’s *t*-test or one-way analysis of variance under SPSS software. A *p* < 0.05 was considered statistically significant. All data generated or analyzed during this study are included in this published article.

## RESULTS

### TPRG1 was upregulated in cystitis glandularis tissues

**FIGURE 1:**
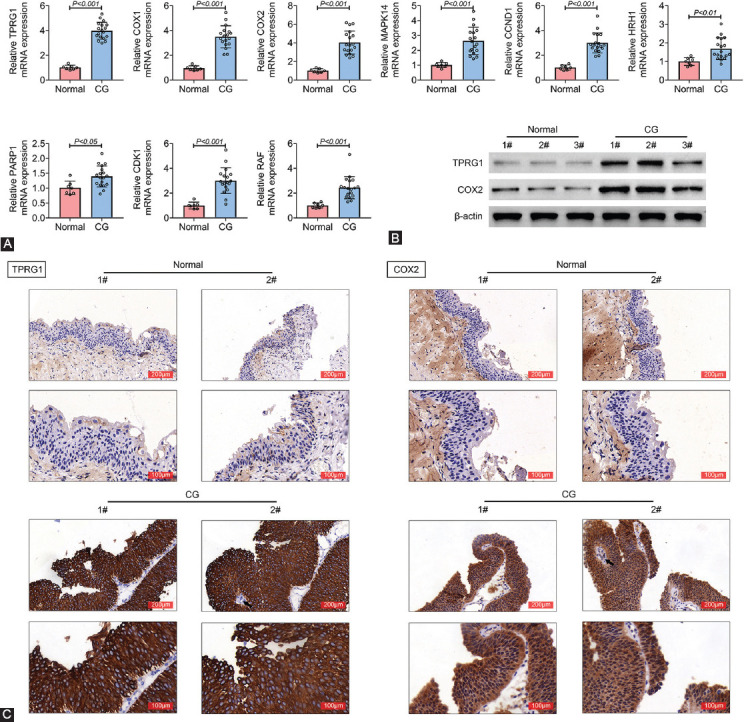
TPRG1 was upregulated in cystitis glandularis tissues. (A) mRNA expression of TPRG1, COX1, COX2, MAPK14, CCND1, HRH1, PARP1, CDK1, and RAF were highly expressed in the CG group compared with that in normal bladder specimen obtained from patients undergoing transurethral bladder tumor resection (Normal group). (B) Protein expression of TPRG1 and COX2 were highly expressed in CG group compared to the normal group. (C) Immunohistochemical analysis confirmed the upregulation of TPRG1 and COX2 in the CG group compared to the normal group. CG: cystitis glandularis.

To investigate role of TPRG1 in cystitis glandularis, we isolated bladder tissues from patients with cystitis glandularis and named as CG group. The mRNA expression of TPRG1, cyclooxygenase (COX)1, COX2, mitogen-activated protein kinase (MAPK) 14, cyclin D1 (CCND1), histamine receptor H1 (HRH1), poly(ADP-ribose) polymerase 1 (PARP1), cyclin-dependent kinase 1 (CDK1) and Raf-1 Proto-Oncogene, Serine/Threonine Kinase (RAF) was increased in the CG group compared with normal bladder specimens obtained from patients undergoing transurethral bladder tumor resection (Normal group) (Figure 1A). TPRG1 and COX2 with the highest expression were performed with the subsequent experiments. CG group also expressed higher protein of TPRG1 and COX2 than the normal group (Figure 1B). Immunohistochemical analysis also confirmed the upregulation of TPRG1 and COX2 in the CG group (Figure 1C), suggesting the possible association between TPRG1 or COX2 and cystitis glandularis.

### TPRG1 was up-regulated in cystitis tissues of E. coli-induced rat

**FIGURE 2:**
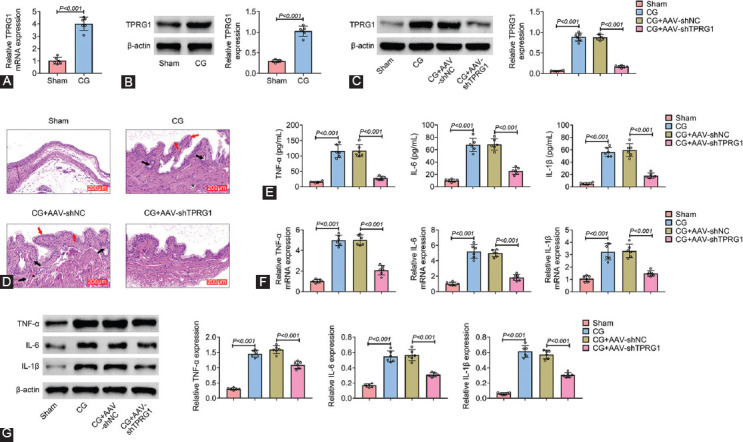
TPRG1 was up-regulated in cystitis tissues of *Escherichia coli -*induced rat. (A) mRNA expression of TPRG1 was up-regulated in *E. coli-*induced cystitis rat compared to the sham-operated rats. (B) Protein expression of TPRG1 was up-regulated in *E. coli-*induced cystitis rat compared to the sham-operated rats. (C) Injection with AAV-shTPRG1 reduced the protein expression of TPRG1 in *E. coli-*induced cystitis rat. (D) Injection with AAV-shTPRG1 reduced the number of Brunn’s nest and cyst numbers and suppressed the inflammatory cell infiltration in *E. coli-*induced cystitis rats. Black arrow refers to Brunn’s nest, red arrow refers to the denuded uroepithelium, and * refers to severe vasocongestion. (E) Injection with AAV-shTPRG1 attenuated *E. coli-*induced up-regulation of TNF-α, IL-6, and IL-1β in cystitis rats detected by ELISA. (F) Injection with AAV-shTPRG1 attenuated *E. coli-*induced up-regulation of TNF-α, IL-6, and IL-1β in cystitis rats detected by qRT-PCR. (G) Injection with AAV-shTPRG1 attenuated *E. coli-*induced up-regulation of TNF-α, IL-6, and IL-1β in cystitis rats detected by western blot.

We then established a rat model with cystitis glandularis through infusion of *E. coli*. Expression of TPRG1 was also up-regulated in *E. coli-*induced cystitis rat compared with the sham-operated rats (Figure 2A and B). The *E. coli-*induced cystitis rats were then delivered with adeno-associated virus (AAV)-mediated silencing of TPRG1. Injection with AAV-shTPRG1 reduced the protein expression of TPRG1 (Figure 2C). H&E staining showed that the bladder mucosa with an identical size of epithelial cells layers was normal in the sham-operated rats and the submucosal layer showed no inflammatory cellular infiltrates and heterocysts (Figure 2D). However, CG group showed significant loss of epithelium and severe vasocongestion in the lamina propria of the bladders (Figure 2D). Brunn’s nests were observed in rats post *E. coli* infusion (Figure 2D) and inflammatory cell infiltration was observed in *E. coli-*induced cystitis rats (Figure 2D). Moreover, injection with AAV-shTPRG1 reduced the number of Brunn’s nest and cyst numbers and suppressed the inflammatory cell infiltration in *E. coli-*induced cystitis rats (Figure 2D). *E. coli-*induced up-regulation of TNF-α, IL-6, and IL-1β in cystitis rats were reversed by AAV-shTPRG1 injection (Figure 2E-G), demonstrating that TPRG1 contributed to inflammation in *E. coli*-induced cystitis rat.

### TPRG1 promoted cell proliferation and migration of primary cystitis glandularis cells

**FIGURE 3:**
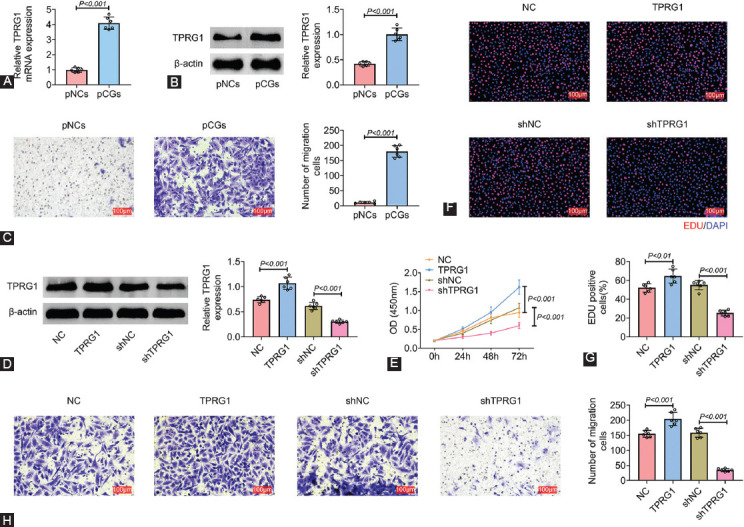
TPRG1 promoted cell proliferation and migration of primary cystitis glandularis cells. (A) mRNA expression of TPRG1 was highly expressed in pCGs compared to pNCs. (B) Protein expression of TPRG1 was highly expressed in pCGs compared to pNCs. (C) PCGs showed higher metastatic ability than the pNCs. (D) Transfection with pcDNA-TPRG1 or shTPRG1 up-regulated or down-regulated TPRG1 expression in pCGs. (E) Over-expression of TPRG1 increased cell viability of pCGs, silence of TPRG1 decreased cell viability of pCGs. (F) Over-expression of TPRG1 increased cell proliferation of pCGs, silence of TPRG1 decreased cell proliferation of pCGs. (G) The relative number of EDU positive cells in pCGs transfected with pcDNA-TPRG1 or shTPRG1. (H) Over-expression of TPRG1 increased cell invasion of pCGs, silence of TPRG1 decreased cell invasion of pCGs. pCGs: primary cystitis glandularis cells. pNCs: primary cells from the normal urinary bladder tissue.

The pCGs and pNCs cells were isolated to investigate role of TPRG1 in cystitis glandularis. TPRG1 was highly expressed in pCGs compared with pNCs (Figure 3A and B). The metastatic ability of pCGs was determined by transwell assay (Figure 3C). pCGs were then transfected with pcDNA-TPRG1 or shTPRG1. Transfection with pcDNA-TPRG1 or shTPRG1 up-regulated or down-regulated TPRG1 expression in pCGs (Figure 3D). Over-expression of TPRG1 increased cell viability of pCGs (Figure 3E) and promoted cell proliferation (Figure 3F and G) and migration (Figure 3H). However, silencing of TPRG1 decreased cell viability of pCGs (Figure 3E) and reduced the number of EdU positive cells (Figure 3F and G) and migration cells (Figure 3H) in pCGs. These results revealed that TPRG1 contributed to cell proliferation and migration capacities of pCGs.

### TPRG1 regulated NF-КB/COX2/PGE2 axis in E. coli-induced cystitis rat

**FIGURE 4:**
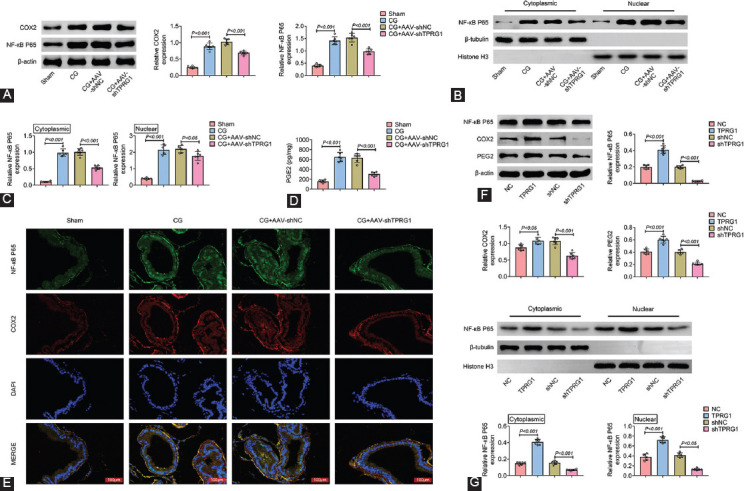
TPRG1 regulated NF-КB/COX2/PGE2 axis in *Escherichia coli -*induced cystitis rat. (A) Injection with AAV-shTPRG1 attenuated *E. coli-*induced up-regulation of NF-КB and COX2 in cystitis rats. (B) Injection with AAV-shTPRG1 attenuated *E. coli-*induced up-regulation of nuclear and cytoplasmic NF-КB in cystitis rats. (C) Relative nuclear and cytoplasmic NF-КB expression in *E. coli-*induced cystitis rats with or without AAV-shTPRG1 injection. (D) Injection with AAV-shTPRG1 attenuated *E. coli-*induced up-regulation of serum level of PGE2 in cystitis rats. (E) Immunofluorescence confirmed that injection with AAV-shTPRG1 attenuated *E. coli-*induced up-regulation of NF-КB and COX2 in cystitis rats. (F) Over-expression of TPRG1 enhanced NF-КB, COX2 and PGE2 in the pCGs, while silence of TPRG1 reduced the expression. (G) Over-expression of TPRG1 enhanced nuclear and cytoplasmic expression of NF-КB in the pCGs, while silence of TPRG1 reduced the expression.

Protein expression levels of NF-КB and COX2 were enhanced in *E. coli-*induced cystitis rats compared with the sham-operated rats (Figure 4A), while injection with AAV-shTPRG1 reduced the expression of NF-КB and COX2 (Figure 4A). The expression of NF-КB in both nuclear and cytoplasmic was increased in *E. coli-*induced cystitis rats, which was decreased by injection with AAV-shTPRG1 (Figure 4B and C). Moreover, injection with AAV-shTPRG1 attenuated *E. coli-*induced the increase in serum level of PGE2 in cystitis rats (Figure 4D). Immunofluorescence staining also confirmed that the up-regulation of NF-КB and COX2 in *E. coli-*induced cystitis rats was restored by AAV-shTPRG1 injection (Figure 4E). Over-expression of TPRG1 enhanced NF-КB, COX2 and PGE2 in the pCGs, while silencing of TPRG1 reduced the expression (Figure 4F). The nuclear and cytoplasmic expression of NF-КB in pCGs were enhanced by TPRG1 over-expression, whereas reduced by TPRG1 knockdown (Figure 4G). This indicatest TPRG1 contributed to activation of NF-КB/COX2/PGE2 axis in *E. coli-*induced cystitis rat.

### Silencing of COX2 attenuated TPRG1-induced increase of cell proliferation and migration of primary cystitis glandularis cells

**FIGURE 5:**
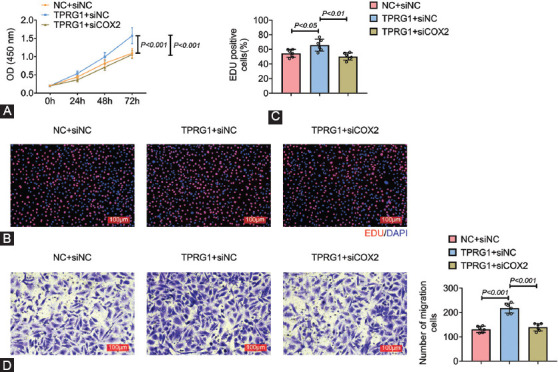
Silence of COX2 attenuated TPRG1-induced increase of cell proliferation and migration of primary cystitis glandularis cells. (A) Silence of COX2 attenuated TPRG1 over-expression-induced increase of cell viability in pCGs. (B) Silence of COX2 attenuated TPRG1 over-expression-induced increase of cell proliferation in pCGs. (C) Relative EdU positive cells in pCGs transfected with pcDNA-TPRG1 or cotransfected with pcDNA-TPRG1 and siCOX2. (D) Silence of COX2 attenuated TPRG1 over-expression-induced increase of cell invasion in pCGs.

The pCGs were cotransfected with pcDNA-TPRG1 and siCOX2 to investigate role of TPRG1/COX2 axis in cystitis glandularis. Increased cell viability of pCGs driven by pcDNA-TPRG1 was decreased by the knockdown of COX2 (Figure 5A). TPRG1 over-expression-induced the increased EdU positive numbers was reversed by interference of COX2 (Figure 5B and C). Silencing of COX2 also attenuated TPRG1-induced increase in cell migration in pCGs (Figure 5D), indicating that TPRG1 contributed to cell proliferation and migration capacities of pCGs through up-regulation of COX2.

## DISCUSSION

The incidence of cystitis glandularis, a proliferative disorder of urinary bladder, is increasing in recent years [13]. Moreover, cystitis glandularis is devoid of effective treatment due to the controversial etiology and pathogenesis [14]. TPRG1 was up-regulated in bladder mucosa isolated from cystitis glandularis patients [10]. This study found that interference of TPRG1 suppressed cell proliferation and migration of human primary cystitis glandularis cells, and ameliorated histopathological changes in bladder mucosa of cystitis rat, thus providing a potential target for the prevention of cystitis glandularis.

Microarray analysis in a previous study has shown that TPRG1 was up-regulated in bladder mucosa of cystitis glandularis patients compared with the normal tissues [10]. We also confirmed the up-regulation of TPRG1 in human bladder mucosa of cystitis glandularis patients through qRT-PCR, western blot and immunohistochemical analyses. Characteristic pathological phenomena, including lymphocyte infiltration, secretory cyst development, and increase in Brunn’s nests, are the main diagnostic strategies for cystitis glandularis [12]. Relation between TPRG1 expression and clinicopathological features of patients with cystitis glandularis should be investigated in further research, which might provide a potential diagnostic biomarker for cystitis glandularis.

Although the pathogenesis of cystitis glandularis remains ambiguous, chronic inflammation or irritation are considered to be the major risk factors for cystitis glandularis [15]. Infection leads to primary irritation of the urothelium, and long-term chronic infection induces chronic inflammation of bladder mucosa, thus resulting in glandular metaplasia of the transitional epithelial cells and hyperproliferative disorder of bladder [16]. Suppression of chronic inflammation and hyperproliferative disorder of bladder contributed to the amelioration of cystitis glandularis [12]. A previous study has shown that TPRG1 was up-regulated in patients with inflammation-related diseases, and TPRG1 stimulated inflammatory responses [8]. Moreover, TPRG1 was considered as an immune-related gene, and correlated with tumor recurrence of stage Ia-b lung cancer [9]. Our results showed that the expression levels of TNF-α, IL-6, and IL-1β were enhanced in *E. coli-*induced cystitis rat, and knockdown of TPRG1 suppressed the inflammatory response in *E. coli-*induced cystitis rat through down-regulation of TNF-α, IL-6, and IL-1β. The cell proliferation capacity of human pCGs was reduced by silencing of TPRG1, suggesting the anti-proliferative and anti-inflammatory effects of TPRG1 silencing against cystitis glandularis. The migration of primary cystitis glandularis cells was also implicated in the pathogenesis of cystitis glandularis [17], and prevention of cell migration in pCGs also represented a potential strategy for cystitis glandularis [18]. Knockdown of TPRG1 in this study suppressed cell migration of human pCGs, thus alleviating cystitis glandularis.

COX-2 is induced by tumor promoters, growth factors, or cytokines at the sites of inflammation, and is involved in the synthesis of prostaglandins and other inflammatory mediators [19]. For example, COX-2 regulated production of PGE2, and reduced COX-2 expression decreased secretion of PGE2 to suppress inflammatory condition in adipocytes [20]. PGE2 increased baseline tension and contractile frequency of urinary bladder urothelium with lamina propria (PGE2 and F2alpha Modulate Urinary Bladder Urothelium, Lamina Propria and Detrusor Contractility through the FP Receptor). Therefore, COX-2 might mediate inflammatory condition in urinary bladder urothelium with lamina propria through regulation of PGE2. Moreover, COX2/PGE2 axis also contributed to the tumor progression [21] and hepatic inflammation [22]. Immunohistochemical analysis has shown that COX-2 was highly expressed in cystitis glandularis tissues compared with the normal tissues [2,23]. Our results also demonstrated the up-regulation of COX-2 in human cystitis glandularis tissues and *E. coli-*induced cystitis rats. Moreover, NF-κB is a critical signaling in inflammatory response, and translocation of NF-κB p65 into nuclear promoted the expression of COX-2 and production of PGE2 in bladder tissues [24]. Block of the NF-κB signaling reduced COX-2 expression [25], and down-regulation of COX-2 and NF-КB enhanced the anti-inflammatory effect against complete freund’s adjuvant-induced arthritis [26]. Our results indicated that level of nuclear NF-κB p65 was significantly up-regulated in *E. coli-*induced cystitis rats, and knockdown of TPRG1 reduced the levels of COX-2 and nuclear NF-κB p65 in *E. coli-*induced cystitis rats and human pCGs. Moreover, PGE2 in human pCGs was also decreased by TPRG1 silencing. Functional assays showed that knockdown of COX-2 attenuated TPRG1 over-expression-induced increase in cell viability, proliferation and migration in human pCGs. Therefore, TPRG1 contributed to cell proliferation and migration of primary cystitis glandularis cells through up-regulation of NF-КB/COX2/PGE2 axis.

## CONCLUSION

We displayed that TPRG1 was involved in inflammation, cell proliferation and migration of cystitis glandularis. Knockdown of TPRG1 suppressed inflammation in *E. coli-*induced cystitis rats, and reduced cell proliferation and migration of human pCGs through down-regulation of NF-КB/COX2/PGE2 axis. Therefore, TPRG1 might be a novel target for the treatment of cystitis glandularis. However, the role of TPRG1/NF-КB/COX2/PGE2 axis in inflammation of pCGs should be investigated in further research.
